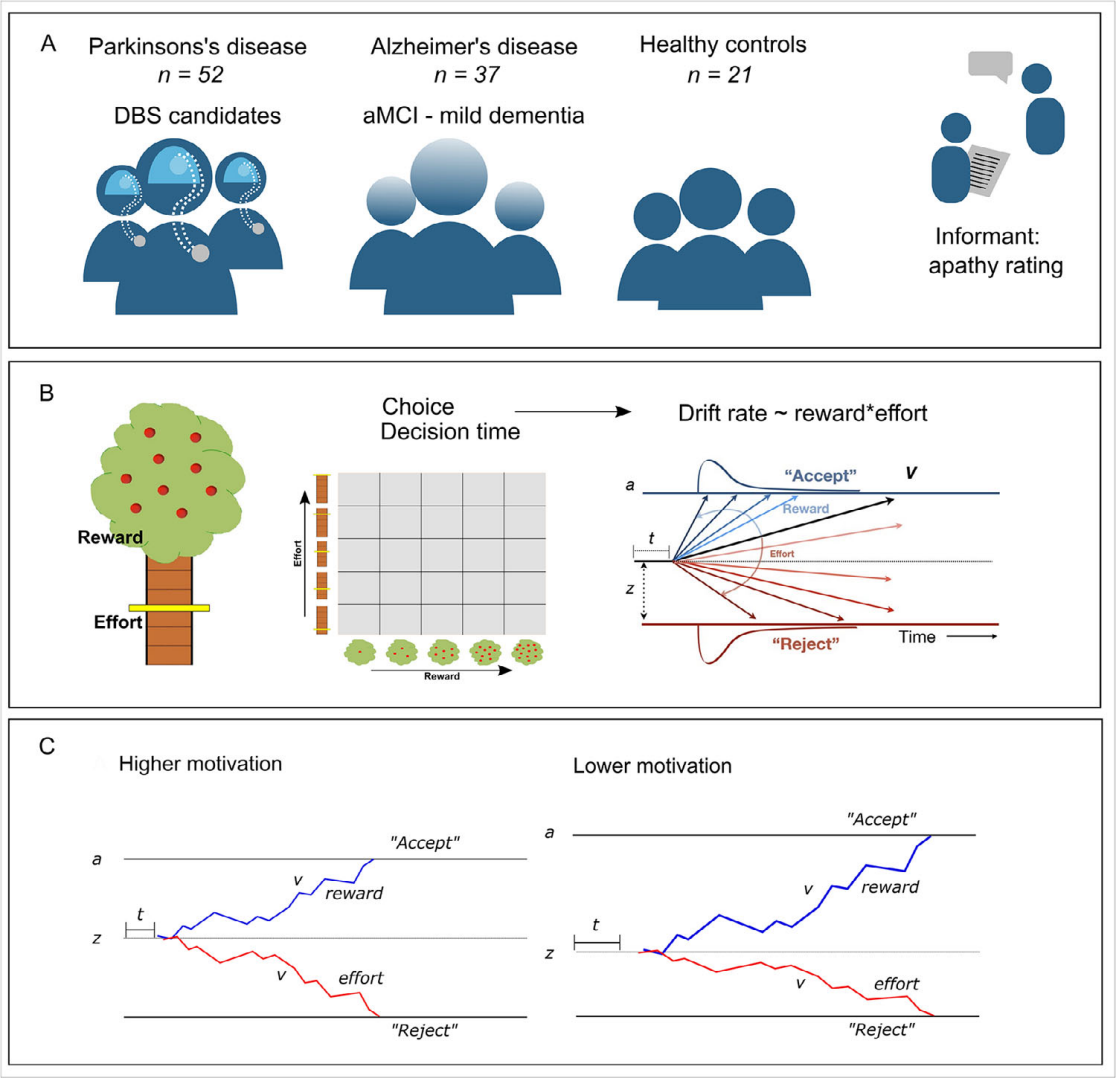# Amotivation associated with altered latent effort‐based decision‐making processes in Alzheimer's and Parkinson's disease

**DOI:** 10.1002/alz70857_105243

**Published:** 2025-12-25

**Authors:** Lee‐Anne Morris, Hideo Suzuki, Seonjoo Lee, Zekai Jin, Yunglin Gazes, Edward D. Huey, Bryan Chen, Ana Marin, Sarah Heibronner, Campbell J Le Heron, Nora Vanegas

**Affiliations:** ^1^ University of Otago, Christchurch, Canterbury, New Zealand; ^2^ Baylor College of Medicine, Houston, TX, USA; ^3^ Mailman School of Public Health, Columbia University, New York, NY, USA; ^4^ Columbia University Irving Medical Center, New York, NY, USA; ^5^ Cognitive Neuroscience Division, Columbia University, New York, NY, USA; ^6^ Department of Psychiatry and Human Behavior, Alpert Medical School of Brown University, Providence, RI, USA; ^7^ University of Otago, Christchurch, New Zealand

## Abstract

**Background:**

Loss of motivation is a prominent syndrome accompanying both Alzheimer's and Parkinson's disease. One approach to understanding motivational loss is to examine the processes involved in the expression of goal‐directed behaviour. Weighing up rewarding outcomes against the effort costs required to obtain them – effort‐based decision‐making – is a core computation when deciding to act for outcomes. Whilst a body of evidence points to disruption of this computation in people with altered motivation, which underlying decision parameters drive this disruption, and whether these would generalise across brain disorders has not been examined. In the current study we examined the latent cognitive processes underlying effort‐based decision making, across a spectrum of motivation and diagnostic groups.

**Methods:**

People with Alzheimer's disease (*n* = 37), Parkinson's disease (*n* = 52) and healthy controls (*n* = 21) performed a physical effort‐based decision‐making task (Apple Gathering Task) and caregiver‐rated apathy scores were recorded. Choices made and reaction times were analysed using drift diffusion modeling to uncover latent cognitive processes. Associations between apathy, diagnosis and latent cognitive processes were examined using linear regression models, controlling for age and cognition.

**Results:**

Across all participants, lower motivation was associated with reduced acceptance of offers to work for reward. Specifically, slower overall drift rate, greater decision threshold, decision bias towards rejecting offers and longer non‐decision time were significantly associated with reduced motivation. Unrelated to motivation, people with AD were less sensitive to changing effort levels, and had a larger decision threshold than PD and control groups.

**Conclusion:**

Loss of motivation in AD and PD is associated with disrupted effort‐based decision‐making processes, including speed of information accumulation, distance between decision options, information encoding and response execution, and bias to choose to do nothing. In addition, AD more generally is associated with greater decision noise, and reduced ability to integrate effort information on choices. In sum, decision‐making tasks are a promising method to investigate the cognitive processes underlying motivational loss in neurodegeneration.